# CBD binding domain fused γ-lactamase from *Sulfolobus solfataricus* is an efficient catalyst for (-) γ-lactam production

**DOI:** 10.1186/1472-6750-14-40

**Published:** 2014-05-13

**Authors:** Jianjun Wang, Junge Zhu, Cong Min, Sheng Wu

**Affiliations:** 1 State Key Laboratory of Microbial Resources, Institute of Microbiology, Chinese Academy of Sciences, Beijing 100101, PR China

**Keywords:** Avicel, Cellulose binding domain, γ-lactamase, (-) γ-lactam

## Abstract

**Background:**

γ-lactamase is used for the resolution of γ-lactam which is utilized in the synthesizing of abacavir and peramivir. In some cases, enzymatic method is the most utilized method because of its high efficiency and productivity. The cellulose binding domain (CBD) of cellulose is often used as the bio-specific affinity matrix for enzyme immobilization. Cellulose is cheap and it has excellent chemical and physical properties. Meanwhile, binding between cellulose and CBD is tight and the desorption rarely happened.

**Results:**

We prepared two fusion constructs of the γ-lactamase gene *gla*, which was from *Sulfolobus solfataricus* P2. These two constructs had Cbd (cellulose binding domain from *Clostridium thermocellum*) fused at amino or carboxyl terminus of the γ-lactamase. These two constructs were heterogeneously expressed in *E. coli* rosetta (DE3) as two fusion proteins. Both of them were immobilized well on Avicel (microcrystalline cellulose matrix). The apparent kinetic parameters revealed that carboxyl terminus fused protein (Gla-linker-Cbd) was a better catalyst. The *V*_max_ and *k*_cat_ value of Avicel immobilized Gla-linker-Cbd were 381 U mg^-1^ and 4.7 × 10^5^ s^-1^ respectively. And the values of the free Gla-linker-Cbd were 151 U mg^-1^ and 1.8 × 10^5^ s^-1^ respectively. These data indicated that the catalytic efficiency of the enzyme was upgraded after immobilization. The immobilized Gla-linker-Cbd had a 10-degree temperature optimum dropping from 80°C to 70°C but it was stable when incubated at 60°C for 48 h. It remained stable in catalyzing 20-batch reactions. After optimization, the immobilized enzyme concentration in transformation was set as 200 mg/mL. We found out that there was inhibition that occurred to the immobilized enzyme when substrate concentration exceeded 60 mM. Finally a 10 mL-volume transformation was conducted, in which 0.6 M substrate was hydrolyzed and the resolution was completed within 9 h with a 99.5% ee value.

**Conclusions:**

Cellulose is the most abundant and renewable material on the Earth. The absorption between Cbd domain and cellulose is a bio-green process. The cellulose immobilized fusion Gla exhibited good catalytic characters, therefore we think the cellulose immobilized Gla is a promising catalyst for the industrial preparation of (-) - γ-lactam.

## Background

γ-lactamase (EC 3.5.2.-) is a small enzyme sub-classification in amidase [1], which could be used for the resolution of (*rac*)-2-azabicyclo [2.2.1] hept-5-en-3-one (γ-lactam) [2]. The well-known utilizations of (-) γ-lactam are in the synthesizing of abacavir [3] and peramivir [4]. These two drugs were applied in the treatment for HIV, hepatitis and influenza pandemic and have brought profits of several billions U.S dollars for the inventors and developers [5]. γ-Lactam could be applied in the syntheses of other leading compounds and drugs too, such as MK-0812 [6], piperidinium compound [7] and melogliptin [8]. In the three preparation methods for the optically pure enantiomers of γ-lactam [1,9-11], the enzymatic method is the most often utilized method because of its high efficiency and productivity. The method was first reported by Taylor et al. [11]. In their work, microbial whole cells were used in bio-enzymatic resolution of racemic γ-lactam. Later, Littlechild and coworkers cloned and purified a (-)-γ-lactamase from *Aurobacterium sp*. and elucidated its catalytic mechanism by protein crystal structure resolution [12]. Up to now four (+) -γ-lactamases have been reported in the forms of *E. coli* recombinant proteins [1,12-14], and others are used in forms of whole cells [11,15,16].

In our previous research works, we immobilized a (+) γ-lactamase (Gla) from *Sulfolobus solfataricus* fused with a His-tags on nickel-chelating agarose and applied the immobilized enzyme in batch reactions. However, agarose is a fragile material and is much more expensive than other immobilizing matrices such as resins. These disadvantages of the agarose-immobilized enzyme confined its usage in scale application. Recently, the cellulose binding domain (CBD) of cellulose is often used as the bio-specific affinity matrix for enzyme immobilization. Compared with agarose, cellulose is much cheaper, and it has excellent chemical and physical properties. More importantly, binding between cellulose and CBD is tight and the desorption rarely happened [17]. Considering these merits of the cellulose immobilized enzyme, we wonder a cbd-fused-gla, immobilized to cellulose, represent a promising catalyst for the resolution of racemic gamma lactam?

In this paper, we developed a method in which the CBD fusion Gla was immobilized on the microcrystalline cellulose matrix (Avicel). We found that the fusion enzyme which had CBD fused at the C terminus was immobilized well on the cellulose matrix, and the immobilized enzyme was more stable than the free fusion enzyme in terms of thermo-stability. The immobilized enzyme retained most of its activity and had a relatively constant efficiency when used for batch transformations. In addition, γ-lactamase showed absolute enantioselectivity to (+)-γ-lactam. In conclusion, we believe that the fusion enzyme immobilized on cellulose, reported in this paper, is a promising catalyst for the industrial preparation of (-)- γ-lactam.

## Methods

### Bacterial strains, plasmids, and culture media

*Escherichia coli* DH5α (Transgen Biotech, Beijing, PRC) cells were used for the cloning studies. *E. coli* rosetta (DE3) (Novagen, Darmstadt, Germany) cells were used for protein expression. pET30a (+) (Invitrogen, California, USA) was used for standard cloning and expression in *E. coli*. The *E. coli* strains were routinely grown in LB medium or on LB agar plates at 37°C. To select bacterial strains carrying the appropriate recombinant plasmids, 35 μg chloramphenicol mL^-1^ or 50 μg kanamycin mL^-1^ was added to the medium. *S. solfataricus* P2 genomic DNA was obtained from the American Type Culture Collection (ATCC, Manasas, VA; catalog number 35092).

### Extraction and purification of DNA

Plasmids were isolated using the TIANprep Mini Kit (Tiangen, Beijing, PR China) according to the protocol provided by the manufacturer. DNA was purified using the TIANgel Midi Kit (Tiangen, Beijing, PR China) according to the manufacturer’s instructions.

### Gene fusion of gla and cip

The *cbd* encoding gene encoding the partial Cbd domain (from 2113 bp to 2581 bp of gene HF912724.1, [18]) was amplified from genomic DNA of *Clostridium thermocellum* ATCC 27405 (ATCC, Manasas, VA) using primer p1 and p2 with *Nco*I and *BamH*I sites respectively (Table 1). A linker sequence GSAGSA was introduced at the C terminus of *cbd*. The PCR protocol consisted of an initial denaturation step of 5 min at 95°C, followed by 30 cycles of 1 min at 95°C, 1 min at 53°C, 1 min at 72°C and ending with a final elongation step for 10 min at 72°C. PCR was performed with Red-*Pfu* DNA Polymerase (Biocolors, Beijing, China). The PCR product was inserted into the according sites of pET30a and the plasmid was named as pET*cbd*.

**Table 1:** Primers used in this study

**Name**	**Sequence (5′ → 3′)**^***a***^	**Description**
p1 forward	CGCCATGGATTTGAAGGTTGAATTCTAC	For construction of pET*cbd*
p2 reverse	CGGGATCC ***AGCGGAGCCAGCGGAGCC*** CACCGGGTTCTTTACCC	
p3 forward	GCGGATCCATGGGAATTAAGTTACCCACATTG	For construction of pET*cbdgla*
p4 reverse	CGCTCGAGTTTTTTGATTCTCTCAAATACAT	
p5 forward	CGCATATGGGAATTAAGTTACCCACATTG	For construction of pET*gla*
p6 reverse	GCGGATCC ***AGCGGAGCCAGCGGAGCC*** TTTTTTGATTCTCTCAAATACAT	
p7 forward	GCGGATCCAATTTGAAGGTTGAATTCTACAAC	For construction of pET*glacbd*
p8 forward	CGCTCGAG CACCGGGTTCTTTACCC	

^*a*^Restriction sites are underlined, sequence encoding the linker peptide were in italics and in grey shade.

The gene encoding the γ-lactamase Gla (GenBank accession no AF290611) was amplified from genomic DNA of *Sulfolobus solfataricus* P2 using primer p3 and p4 with *BamH*I and *Xho*I sites respectively. (Table 1). The PCR protocol was same as for *cbd.* The PCR product was inserted into the *BamH*I and *Xho*I sites of pET*cbd* and the final plasmid was named as pET*cbdgla*. This plasmid harbored a *cbd*-*linker*-*gla* fusion gene.

In a same way, *gla* was amplified with primer p5 and primer p6 and inserted into the *Nde*I and *BamH*I sites of pET30a (pET*gla*). The linker sequence was introduced at the C terminal of *gla*. Then *cbd* was amplified with primer p7 and p8 and introduced into downstream the linker sequence (*BamH*I and *Xho*I sites). The final plasmid was named as pET*glacbd* and it harbored a *gla*-*linker*-*cbd* fusion gene.

### Expression of fusion gene in *E. coli* rosetta (DE3) cells

*E. coli* rosetta (DE3) was chosen as the expression strain because in the strain Gla showed higher specific activity than in other strains such as *E. coli* (DE3) (data not shown). This is because there were many rare codons existed in *gla* gene. The constructs were transformed into *E. coli* rosetta (DE3) cells and has grown overnight at 37°C. Stock culture (5 mL) was transferred into fresh 500-mL culture medium and has grown up to an optical density of 0.8 (OD 580). Isopropylthiogalactoside (IPTG) was then added to a final concentration of 1.0 mM, and after incubation for 2 h, the cells were harvested by centrifugation (4,000 × *g* for 10 min).

### Purification of Gla fusion proteins on a nickel (Ni)-chelating column

Purification of the γ-lactamase fusion protein on the Ni-chelating column was carried out using the protocol and buffer supplied by Novagen (New Jersey, USA). Cells from the expression culture (500 mL) were suspended in 40 mL binding buffer (50 mM Tris–HCl, 300 mM NaCl, and 10 mM imidazole, pH 8.0) and disrupted by ultrasonication. The crude samples were heated to 60°C for 10 min then centrifuged. The supernatant was applied to a 1-mL Novagen His•Band gravity flow column that had been equilibrated with 20 mL Ni-NTA binding buffer. The column was then washed with 20 mL wash buffer (50 mM Tris–HCl, 300 mM NaCl, and 20 mM imidazole, pH 8.0). The His-tagged proteins were eluted with 10 mL elution buffer (50 mM Tris–HCl, 300 mM NaCl, and 200 mM imidazole, pH 8.0). To remove the imidazole present in the elution buffer, the eluate was applied to a 5-mL HiTrap desalting column (GE Healthcare, New Jersey, USA) connected to an ÄKTA FPLC system (GE Healthcare), and the column was eluted with 50 mM Tris–HCl buffer (pH 7.5, 150 mM NaCl). The protein samples were collected and dialyzed against 5000 mL of 50 mM Tris–HCl buffer (pH 8.0). The buffer was changed three times over a period of 24 h. The samples were then ready for use.

### Immobilization of the crude or purified γ-lactamase fusion proteins on Avicel

For crude fusion proteins, cells from the expression culture (500 mL) were suspended in 40 mL ultrasonication buffer (50 mM Tris–HCl, pH 8.0) and disrupted by ultrasonication. The ultrasonication sample was heated to 60°C for 10 min then centrifuged (13,000 × *g*, 10 min). 5 mL of supernatant was added with 1 g of Avicel and kept at 25°C for 120 min with stirring. For purified fusion proteins, 1 mL protein solutions (20 mg protein in 50 mM Tris–HCl, pH 8.0) were added with 100 mg Avicel and kept at 25°C for 120 min with stirring. Finally the immobilized fusion proteins were collected by centrifuge (3,000 × *g*, 10 min). From hereafter the mass of the immobilized enzyme was included in Avicel and the mass of the absorbed protein on Avicel. When protein concentration of immobilized enzyme was involved in calculations, only the mass of the absorbed protein was counted.

### Adsorption Isotherm Measurements for crude fusion proteins

Adsorption isotherm measurements were conducted at room temperature (25°C) in 50 mL tubes. Samples containing 2,000 mg of crude fusion proteins (supernatant samples after treated at 60°C, total activity 5500 U) and 1 g of Avicel were mixed in a final aqueous volume of 5 mL. Suspensions were incubated for 120 min to allow the adsorption to saturate. The protein concentration of the supernatant was measured at regular intervals using a BCA protein assay kit (Pierce, USA), and this value was then subtracted from the initial amount of protein and the difference was considered to be amount of proteins adsorbed onto Avicel. Otherwise, the total activity of supernatant was measured and this value was then subtracted from the initial enzyme activity. All values were measured in triplicate.

### Standard reaction conditions and γ-lactamase activity assay

γ-Lactamase activity was assayed by adding 1 mg of enzyme (or 25 mg immobilized enzyme) to 200 μL of the 4.6 mM substrate solution (pH 7.0, 50 mM phosphate buffer). If not specified, the reaction solution was incubated at 70°C for 30 min. After transformation the reaction solution was extracted with 100 μL ethyl acetate and analyzed by chiral HPLC. One unit (U) of enzyme activity was defined as the amount of enzyme that catalyzed the conversion of one micromole of substrate per minute. As to immobilized enzyme the absorbed protein was first calculated based on the binding capacity described above, then the activity was determined.

### Temperature optimum and temperature stability determination

For temperatue optimum determination, the transformation solutions (free enzyme) or suspensions (immobilized enzyme) as described in standard reaction conditions were incubated and reacted at various temperature from 20°C to 90°C with a 10 degree interval. Then the samples were analyzed as described in standard reaction conditions and activity assay.

For temperature stability determination, 50 μL of enzyme solution (1 mg enzyme in pH 7.0, 50 mM phosphate buffer) or 100 μL of immobilized enzyme suspension (25 mg immobilized enzyme, pH 7.0, 50 mM phosphate buffer) were incubated at various temperature from 20°C to 90°C with a 10 degree interval for 30 min. Then the enzyme solution or suspension was added to the substrate solution as described in standard reaction conditions. The samples were allowed to react and later analyzed as described in standard reaction conditions and activity assay.

### Analytical chiral HPLC

The reaction solution (200 μL) was extracted with 100 μL ethyl acetate, and benzamide was added to the extract as the internal standard. The ethyl acetate extract (10 μL) was applied to a Daicel Chiralpak AS-H column (Daicel, Tokyo, Japan) and eluted with a mobile phase consisting of 80% acetonitrile and 20% isopropanol (volume ratio). The UV absorbance of eluted γ-lactam was measured at 230 nm. Benzamide, (+) γ-lactam, and (-) γ-lactam had retention times of 8.7, 11.8, and 13.9 min, respectively, at a flow rate of 0.6 mL/min.

### Determination of the apparent kinetic parameters

The apparent kinetic parameters were determined by the Lineweaver-Burk plot. Enzymatic reactions were carried out with substrate solutions of different concentrations (ranging from 4.6 mM to 32.2 mM). To ensure accuracy, substrate conversion was controlled below 10%.

### Protein assay and sodium dodecyl sulfate-polyacrylamide gel electrophoresis (SDS-PAGE)

The protein concentration was determined by the BCA Protein Assay Kit from Pierce (Rockford, USA) using bovine serum albumin (BSA) as the standard. SDS-PAGE was performed as described earlier using a 6% stacking gel and 12% separating gel [19].

### DNA sequencing

DNA sequencing reactions were performed on a plasmid template using the ABI Prism BigDye terminator cycle sequencing ready reaction kits (Applied Biosystems, California, USA) and an ABI PRISM 377 DNA sequencer (Applied Biosystems).

### Software and online service

The BLAST × [20] program was used for protein homology search.

## Results and discussion

### Expression and purification of γ-lactamase fusion proteins

**Figure 1:**
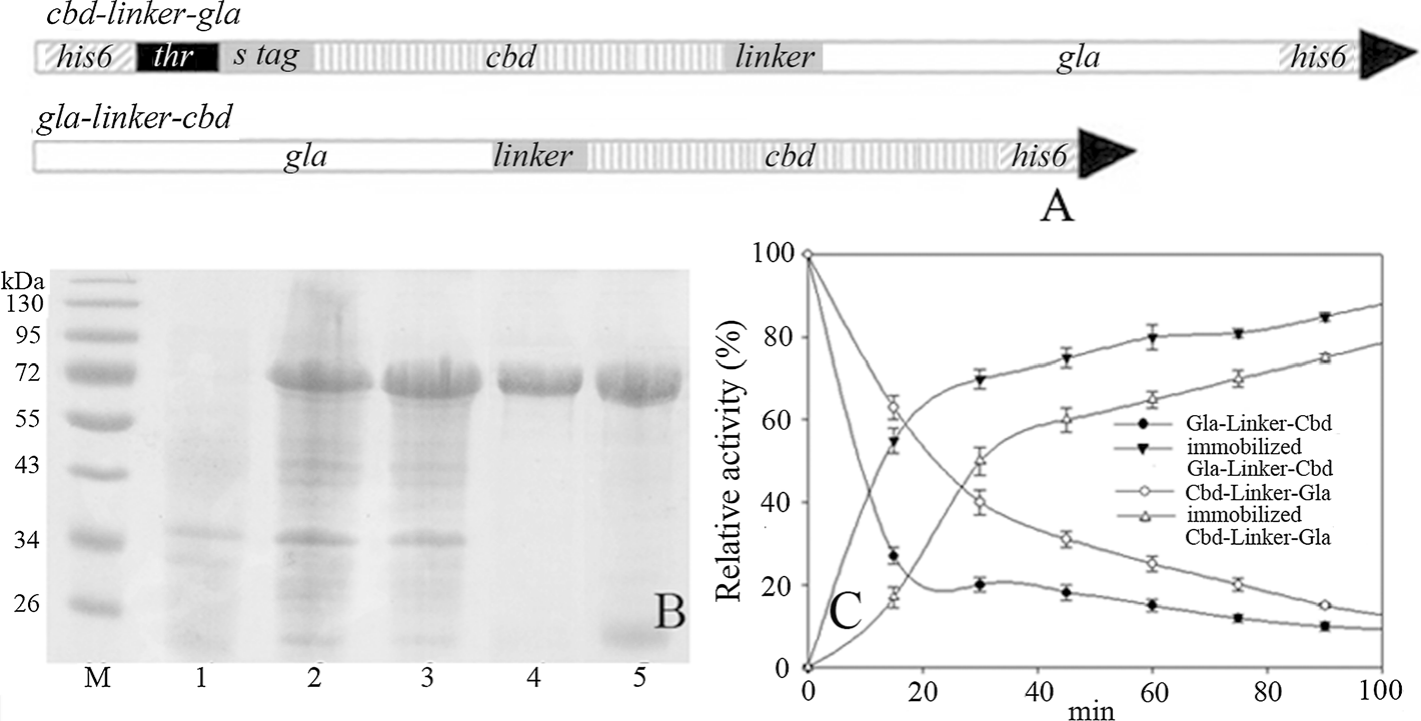
**Construction of *cbd-linker-gla *and *gla-linker-cbd *fusion genes (A) and expression of the two constructs (B) and adsorption isotherm measurements for crude fusion proteins (C).** A. his6: his-tag encoding gene; thr and s tag: thrombin cleavage site and S peptide tag on pET30; B. 1.*E. coli* rosetta (DE3) harboring pET30; 2.*E. coli* rosetta (DE3) harboring pET*cbdgla*; 3.*E. coli* rosetta (DE3) harboring pET*glacbd*; 4.Purified Cbd-Linker-Gla; 5.Purified Gla-Linker-Cbd C. The total activity of Gla-Linker-Cbd supernatant at 0 h (5500 U) was set as 100% relative activity.

The γ-lactamase structural gene was a 1,515-bp fragment. The *cbd* encoding gene was a 469-bp fragment [18] and the linker sequence was an 18-bp fragment. The two fusion gene constructs were expressed as two separate proteins respectively. The *cbd*-*linker*-*gla* construct expressed a 78-kDa fusion enzyme that had a 4.7-kDa fusion peptide at its amino (N) terminus and a His-tag at its carboxyl (C) terminus (pET30a). The other construct (*gla* -*linker-cbd*) expressed a 74.2-kDa single fusion enzyme that only had a 0.9-kDa C terminal His-tag (Figure 1A). The recombinant enzymes were expressed in the soluble fraction of the bacterial lysate (Figure 1B lane 2 and 3). After single-step purification on Ni-chelating column, the purified proteins were applied for SDS-PAGE (Figure 1B lane 4 and 5). There was a low-molecular-weight protein contamination in the pure protein of *gla* -*linker-cbd*, therefore in this paper the enzyme activity of *gla* -*linker-cbd* was probably lower than the actual value.

### Comparison of the apparent kinetic parameters

Analysis of the apparent kinetic parameters revealed obvious differences in the *K*_m_, *V*_max_ and *k*_*cat*_ values of the two forms of free fusion γ-lactamase protein. The changes in these values indicated that the N-terminal Cbd fusion enzyme showed lower substrate affinity than the C-terminal Cbd fusion enzyme, and its *V*_max_ and *k*_*cat*_ values were also much lower than the C-terminal fusion enzyme (Table 2). After immobilization the *K*_m_ and *V*_max_ values of both fusion proteins were improved, and their *k*_*cat*_ values were also upgrade significantly. However, there was no obvious difference on enzyme turnover number between the C-terminal Cbd fused enzyme and the N-terminal Cbd fused enzyme.

**Table 2:** Apparent kinetic parameters of the free fusion proteins and their immobilized counterparts

**Fusion protein**	***K***_**m **_**(mmol l**^**-1**^**)**	***V***_**max **_**(U mg**^**-1**^**)**	***k***_***cat ***_**(s**^**-1**^**)**
Cbd-Linker-Gla	14.3 ± 1.6	71.6 ± 4.2	8.8 × 10^4^
Immobilized Cbd-Linker-Gla	34.0 ± 1.6	344.9 ± 31.2	4.2 × 10^5^
Gla -Linker- Cbd	5.7 ± 0.1	151.5 ± 30.3	1.8 × 10^5^
Immobilized Gla -Linker- Cbd	25.0 ± 1.6	381.6 ± 44.2	4.7 × 10^5^

### Adsorption isotherm measurements for crude fusion proteins and purified Gla-Linker-Cbd

To make the method more feasible, first we tried to absorb the crude fusion proteins (supernatants after ultrasonication) on Avicel. The adsorption isotherms of the two crude fusion proteins were measured. However, the protein concentration changes were too small and the experiment errors were far beyond normal values. Therefore, the relative activities of the crude protein supernatants instead of the protein concentration were determined. After absorption the remaining supernatants and the enzyme absorbed Avicels were applied for (*rac*)-γ-lactam transformation at 60°C and then the transformations were analyzed by chiral HPLC respectively. The total activities of the supernatant and immobilized enzyme were calculated. According to Figure 1C, the binding between Gla-Linker-Cbdand Avicel was faster than the binding between Cbd-Linker-Gla and Avicel. The binding was in a stable state after 120 min absorption. The purified fusion proteins were also used in the absorption experiments. Both the adsorption ratios of the two purified proteins on Avicel were 40 ± 6 mg protein per g Avicel. The binding capacity was calculated to be 0.8 ± 0.1 μM/g, which was in the range for CBD-fusion proteins [21-23]. The binding saturation time of the purified protein was same as the crude protein (data not shown). Based on the above experiment result, Gla-Linker-Cbd immobilized protein was chose for further experiments.

### Comparison of the temperature property of free Gla-Linker-Cbd protein and its immobilized counterpart

**Figure 2:**
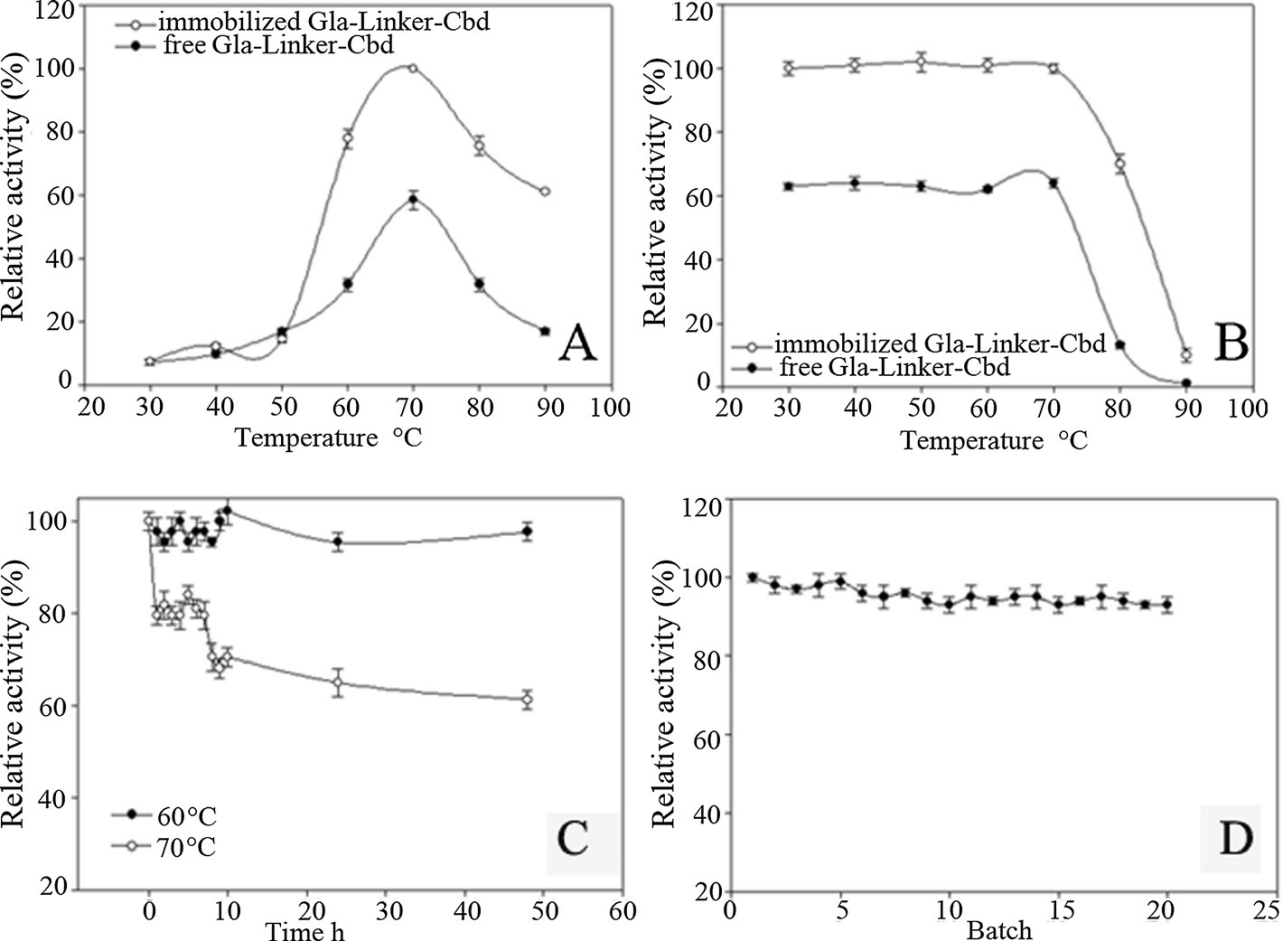
**Comparison of the optimal temperatures (A) and thermostabilities (B) between free Gla-Linker-Cbd protein and its immobilized counterpart, and thermostability of immobilized Gla-Linker-Cbd at 60°C and 70°C (C) and batch stability (D).** A. Optimal temperature; B. Thermostability. The activity of immobilized Gla-Linker-Cbd at 70°C **(A)** or firstly incubated at 30°C for 30 min (B, 145 ± 12 U mg^-1^) was set as 100% relative activity. In B, firstly enzymes were incubated at designated temperature for 30 min then applied for catalysis. The activity of immobilized Gla-Linker-Cbd in cubated at 60°C **(C)** or the first batch (D, 116 ± 6 U mg^-1^) was set as 100% relative activity.

The temperature optima and thermostability of Gla-Linker-Cbd and its immobilized counterpart were determined. The optimal temperature of Gla protein was at 80°C [2]. However, after fused with Cbd the optimal temperatures of the fusion protein and its immobilized counterpart dropped from 80°C to 70°C (Figure 2A). The immobilized enzyme exhibited higher specific activity than the free enzyme. Meanwhile, according to Figure 2B, after incubated at 80°C for 30 min, the fusion protein lost almost 80% of its original activities while its immobilized counterpart only lost 30% of its original activities.

Protein thermostability was often related to protein rigidity [24]. In *C. thermocellum* most of the Cbd fusion enzymes showed maximum activity at 65°C or 75°C, and had weak thermostabilities at 80°C [25-27]. Therefore, compared to Gla the rigidity of the fusion protein probably was reduced. In this balance process Cbd was the determinant part to affect the protein thermostability.

### Stability of immobilized enzymes at 60°C and 70°C

All the other conditions were same as described in standard transformation conditions. The thermostability of the immobilized enzyme was tested at 60°C and 70°C respectively. The immobilized enzyme was stable at 60°C even incubated for 48 h. However, when incubated at 70°C for 1 h the enzyme lost their 20% of the initial activity. If the incubation time extended to 48 h, the immobilized enzyme lost their 40% initial activity (Figure 2C). The batch stability was tested at 60°C for 20 batches reactions. After each transformation, the immobilized enzyme was collected by centrifuge and applied for another transformation. According to Figure 2D, the immobilized enzyme maintained almost 100% of their initial activity after 20 batches reactions.

### Optimization of the concentrations of immobilized enzymes and substrate in batch transformation

**Figure 3:**
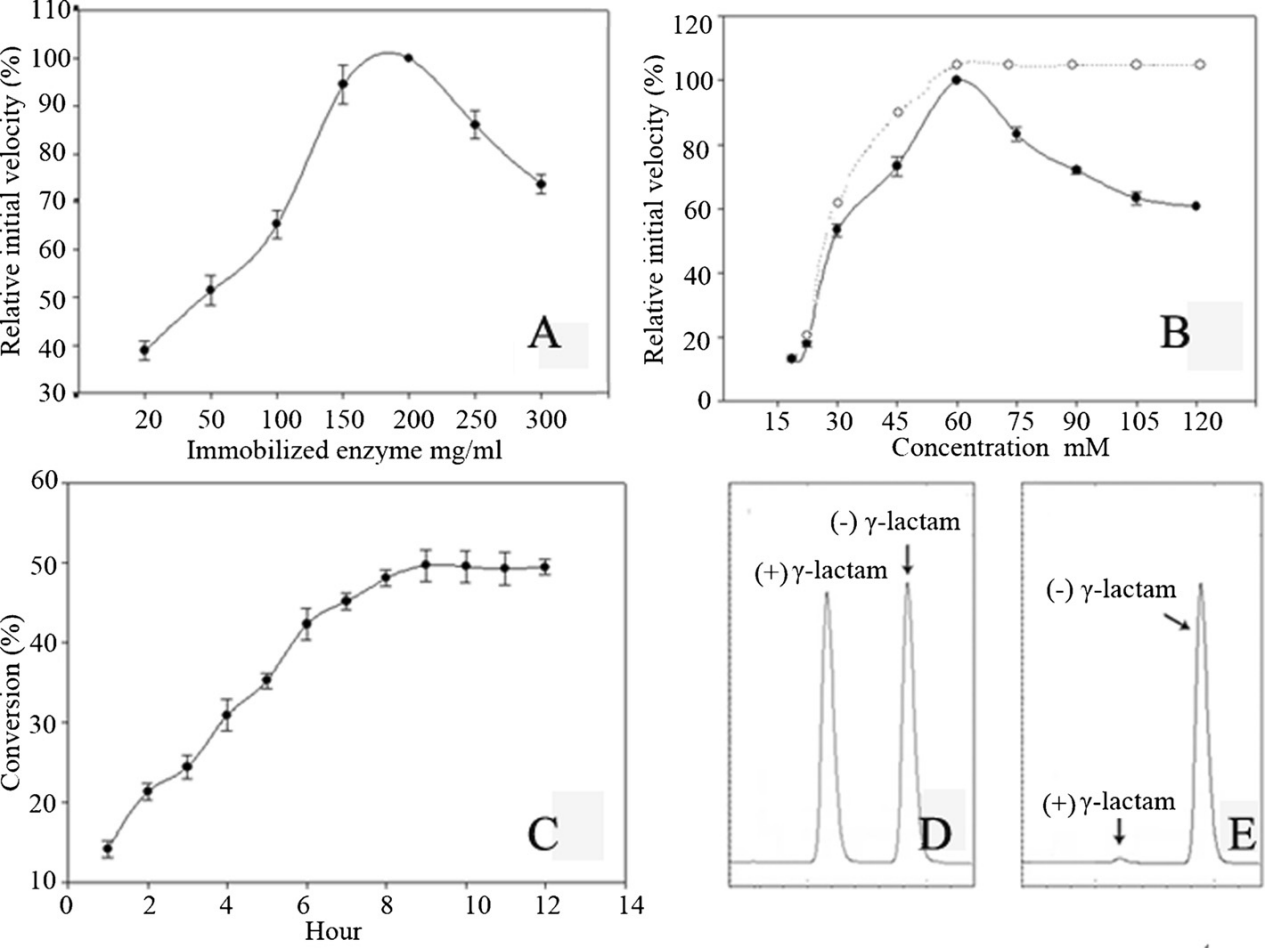
**Optimal concentration of immobilized enzyme (A) and substrate (B), and time course of the optimized transformation (C) and chiral HPLC analyses of the (*rac*)-γ-lactam control (D) and sample after the 9 h transformation (E).** The activity of 200 mg/ml immobilized Gla-Linker-Cbd at 60°C (A, 120.6 ± 5.2 μmol/min) and immobilized Gla-Linker-Cbd to 60 mM substrate (B, 338.6 ± 15.2 μmol/min) was set as 100% relative activity. The line in dot in B represents no substrate inhibition to enzyme. C: Substrate concentration 0.6 M, immobilized enzyme concentration 200 mg/ml, transformation temperature 60°C.

In term of cost saving, in a transformation the substrate concentration should be as high as possible, and the transformation time should be as short as possible. Taking into account of these two factors, we tested first the highest immobilized enzyme concentration employed in the system, and then the highest substrate concentration. In these optimizations, the initial velocity of transformation was used as the response factor. According to Figure 3A, the optimal enzyme amount in the transformation system was 200 mg/mL. Beyond or below this value, the initial velocity of reaction dropped dramatically. It was easy to understand that lower enzyme amount meant lower catalytic activity. On the other hand, 200 mg immobilized enzyme almost had an autogenous volume of 0.5 mL in transformation buffer, and it was impossible to stir the reaction suspension sufficiently in the situation. Therefore the mass transfer efficiency of the system was probably affected severely [28] and the initial velocity of the enzyme was reduced significantly.

The enzyme initial velocity would be upgraded by increasing the substrate amount till it reached and maintained maximum activity if there was no substrate inhibition [29]. According to Figure 3B, when the substrate concentration exceeded 60 mM, the initial velocity of the enzyme dropped obviously, which indicated that substrate inhibition occurred.

Though there is substrate inhibition, it does not mean that higher concentration can’t be applied. We tested the maximum substrate concentration for 200 mg/mL immobilized enzyme in prolonged reaction time. When the substrate concentration was below 0.6 M, the reaction conversion reached 49.8% in various time. If the substrate concentration was beyond this value, the conversion hardly reached 40% even transformed for 96 h, which was very uneconomical (data not shown).

After optimization, we conducted a 10 mL-volume transformation in which 2 g immobilized enzyme was suspended in 0.6 M substrate buffer solution. The reaction was conducted at 60°C and at a certain time interval, 10 μL of sample aliquot was extracted with ethyl-acetate and then applied for chiral HPLC analysis. According to Figure 3C, the transformation was completed within 9 h (49.5% conversion). The *ee* value of the remaining substrate (-) γ-lactam was 99.5% (Figure 3E). The reaction solution was extracted with 30 mL dichloromethane each time for three times. The combined solvent was recovered by using rotary evaporator. At last, 0.30 g (-) γ-lactam in crystal was recovered from the transformation solution, and the recovery rate was 92%.

## Conclusions

In our previous work, we employed a single purification and immobilization method to immobilize *Sulfolobus solfataricus* γ-lactamase on Ni-NTA agarose. The method is simple and easy to conduct. And as we reported previously [2]*V*_max_ and *k*_cat_ value of the agarose immobilized enzyme were around 450 U mg^-1^ and 4.2 × 10^5^ s^-1^ respectively, which indicated it was also an efficient immobilized catalyst same as the cellulose immobilized enzyme. However because of the fragility of agarose, the activity of immobilized enzyme decreased sharply after multiple usages probably due to the structure crash of agarose. It was clear that the decrease did not come from protein inactivation, because under the same condition the enzyme was not deactivated even for a longer time. On the contrary cellulose is a strong material. Therefore it is a very good candidate as immobilizing material also because it is the most abundant and renewable material on the Earth. And most importantly, unlike glutaraldehyde cross-linked immobilization, the absorption between Cbd domain and cellulose is a bio-green process, in which no extra energy or expensive and harming catalyst is needed. Based on the data provided in this paper we think the cellulose immobilized Gla is a promising catalyst for the industrial preparation of (-)-γ-lactam, which is a very important drug intermediate.

## Competing interests

The authors declare that they have no competing interests.

## Authors’ contributions

JW and CM carried out the cloning, over-expression, purified and immobilization. JZ characterized the immobilized enzyme. SW directed the over-all study and drafted the manuscript. All authors read and approved the final manuscript.
